# An Acute Case of Intoxication with Cyanobacteria and Cyanotoxins in Recreational Water in Salto Grande Dam, Argentina

**DOI:** 10.3390/md9112164

**Published:** 2011-10-31

**Authors:** Leda Giannuzzi, Daniela Sedan, Ricardo Echenique, Dario Andrinolo

**Affiliations:** 1 Toxicology Laboratory, Exact Sciences College, National University of La Plata (UNLP), 47th and 115th Street, La Plata 1900, Argentina; E-Mails: danielasedan@yahoo.com.ar (D.S.); dandrinolo@yahoo.com (D.A.); 2 Department of Phycology, Natural Sciences and Museum College, National University of La Plata (UNLP), 47th and 115th Street, La Plata 1900, Argentina; E-Mail: rechen@fcnym.unlp.edu.ar

**Keywords:** cyanobacteria, cyanotoxins, microcystin-LR, acute case

## Abstract

Cyanobacterial blooms and hepatotoxic microcystins (MCs) usually occur in summer, constituting a sanitary and environmental problem in Salto Grande Dam, Argentina. Water sports and recreational activities take place in summer in this lake. We reported an acute case of cyanobacterial poisoning in Salto Grande dam, Argentina, which occurred in January 2007. Accidentally, a young man was immersed in an intense bloom of *Microcystis* spp. A level of 48.6 μg·L^−1^ of microcystin-LR was detected in water samples. Four hours after exposure, the patient showed nausea, abdominal pain and fever. Three days later, dyspnea and respiratory distress were reported. The patient was hospitalized in intensive care and diagnosed with an atypical pneumonia. Finally, a week after the exposure, the patient developed a hepatotoxicosis with a significant increase of hepatic damage biomarkers (ALT, AST and γGT). Complete recovery took place within 20 days. This is the first study to show an acute intoxication with microcystin-producing cyanobacteria blooms in recreational water.

## 1. Introduction

In the past decades, increasing eutrophication has led to frequent outbreaks of cyanobacterial blooms in many freshwater environments of the world. Many cyanobacteria species such as *Microcystis* spp., *Anabaena* spp., *Cylidrospermopsis* spp. are causing serious environmental problems since they are able to produce several natural toxins. Hepatotoxic microcystins (MCs) are the most frequently reported cyanotoxins in eutrophic freshwater bodies.

About 80 MCs isoforms have been isolated and identified, but only a few have been detected in high concentrations, of which MC-LR is the most widely found [1]. Microcystins inhibit serine/threonine-specific protein phosphatases (PPs) such as PP1 and PP2A through the binding to these enzymes [2,3]. The acute toxicity of MC can be explained by this phosphatase inhibition which leads to an excessive phosphorylation of proteins and to alterations in cytoskeleton and loss of cell shape with subsequent destruction of liver cells causing intrahepatic haemorrhage or hepatic insufficiency [4]. Even though outbreaks of human illness associated with cyanobacterial have been sporadically reported for decades, information about clinical signs and symptoms of cyanobacterial toxin poisonings mainly deal with animal poisonings and laboratory studies [5].

A study of recreational exposure to microcystins among 81 children and adults in recreational activities in two California reservoirs, with significant ongoing blooms of toxin-producing cyanobacteria, including *Microcystis aeruginosa,* was reported by Backer *et al*. 2010 [6].

In Argentina, dense cyanobacterial blooms frequently occur in several large reservoirs (San Roque, Cordoba, Salto Grande, Entre Rios) in warm seasons every year.

Salto Grande dam is a shallow eutrophic lake that maintains regular occurrence of cyanobacterial surface blooms, mainly composed of *Microcystis* spp., a frequent producer of MCs. It is used as a water supply, for recreational activities and for fishing. It has been monthly monitored since 2006 by our research team in order to detect cyanobacteria and cyanotoxins [7].

Acute exposures to cyanobacteria and their toxins may occur via oral, dermal, inhalational or intravenous routes. However, the most common exposures are believed to occur during recreational and occupational contact with cyanobacteria in lakes, lagoons and rivers [8]. The unspecific symptoms of sub-acute intoxications and the difficulties to properly recognize the etiology of these symptoms conspire against the ability to diagnose this kind of intoxication [9].

This paper reports an acute case of cyanobacteria poisoning in Salto Grande Dam, Argentina due to an accidental exposure of a young man to a cyanobacterial bloom. We report several biochemical parameter changes found during acute and recovering stages of illness.

### Description of the Case

On January 7th, 2007 a nineteen-year-old man was practicing water sports with his jet ski in Salto Grande dam, Argentina. He accidentally ended up in a bay where there was a big patch of “green paint”, as he described it later. He was immersed in this water for two hours. After that, he swam back to shore, dragging his jet ski. The location coordinates are 30°53′29″S–55°58′05″W. A few hours later, the young man began to experience gastrointestinal malaise, nausea and vomiting and muscle weakness. The first medical diagnosis was idiopathic stress, indicating rest at home. The patient’s condition worsened after four days, requiring hospitalization and intensive medical care. The doctors could not understand the picture or the trend it was taking; a patient with initial pulmonary problems results in a liver disorder.

The patient was admitted to a medical center with dyspnoea, nausea, abdominal pain and fever syndrome with 48 to 72 h of progression after initial exposure. At that moment, respiratory distress, hypoxemia (PO_2_ 40 mmHg, lung infiltrate), renal failure (creatinine 2.4 mg·dL^−1^) decreasing platelets (Plq 40,000 cell·mL^−1^), increasing leukocytes (15,000 cell·mL^−1^) and rise of some liver enzymes (aspartate transaminase (AST) 280 IU·L^−1^, alanine transaminase (ALT) 300 IU·L^−1^, γ-glutamyltransferase (γGT) 280 IU·L^−1^) were detected without abnormal levels of bilirubin (total bilirubin 0.5 mg%) and alkaline phosphatase (ALP).

The patient was mechanically ventilated and an empirical antibiotic treatment (Imipenem and Claritromicine) was given. Both low lobes were observed to have generalized interstitial infiltrate when complementary studies such as chest X-ray and computerized axial tomography lung hepatization were carried out.

Additional blood culture and serum samples were analyzed in order to detect and identify several microorganisms and virus like HIV, Epstein-Barr virus, *Clamidia pneumoniae* and *Mycoplasma*, frequently involved in pulmonary disease. Those results were negative. No alterations were found in abdominal ultrasound scan, electrocardiogram, brain computerized axial tomography or cerebrospinal fluid analysis.

The patient’s condition improved; 72 h after admission, mechanical ventilation was removed and after 8 days he went out of intensive care. All parameters returned to their normal values after 20 days and the patient was discharged without any symptoms. No permanent damage has been observed.

## 2. Results and Discussion

### 2.1. Quantification of Phytoplankton and Microcystin in Water Sample

Total phytoplankton ranged between 33,680 and 35,740 cell·mL^−1^. The most abundant species was *Microcystis wesenbergii*, with values between 30,600 and 31,600 cell·mL^−1^. However, *Microcystis aeruginosa* was detected in the range of 3080–4100 cell·mL^−1^.

**Figure 1:**
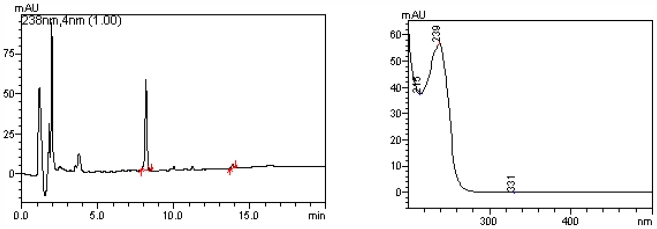
High performance liquid chromatography (HPLC) profile of photodiode array detector (DAD) from microcystin from water samples.

High levels of Microcystin-LR were detected in water samples (48.6 ± 15 μg·L^−1^, *N* = 3). Figure 1 shows a typical elution profile for water sample, with a peak corresponding to microcystin-LR at 8.2 minutes of retention time. The typical Microcystin absorption spectrum for this peak is also shown.

Only one peak was observed in the chromatograms and it had the same retention time of MC-LR.

### 2.2. Acid-Base Status Parameters

**Figure 2:**
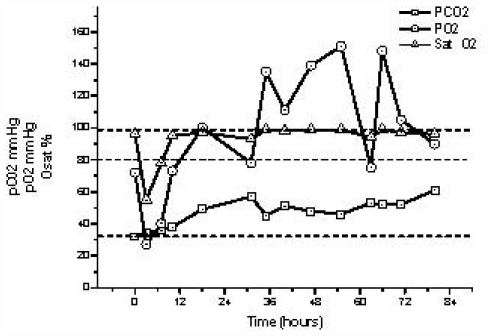
Evolution of acid-base status parameters: □ PCO2 (mmHg); Δ PO2 (mmHg); ○ oxygen saturation (Sat O2) (%). Dotted lines are normal values.

When admitted to the medical center, the patient had a counterbalanced respiratory acidosis, which is characterized by an increase in PCO_2_. Figure 2 shows the evolution of acid-base status parameters. Decreased levels of PO_2_ and oxygen saturation (Sat O_2_) of hemoglobin were observed during first 18 h after admission. During this period the lowest levels were 27 mmHg for PO_2_ and 55% for SaO_2_ at 3 h. Hypoxemia and hypercapnia returned to normal after first critical 72 h. HCO_3_ ^−^ therapy was not necessary in this case.

### 2.3. Liver Injury Biomarkers

**Figure 3:**
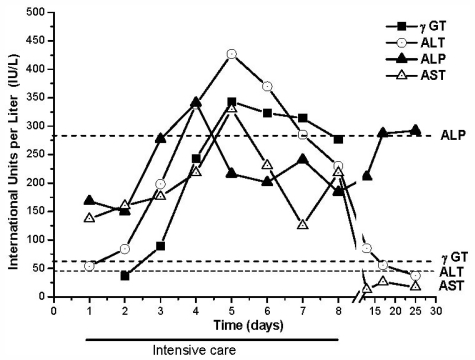
Evolution of liver function parameters in serum samples. ▾ γ-glutamyltransferase (GGT); ○ Alanine aminotransferase (ALT) (IU.L-1); ▴ Alkaline phosphatase (ALP) (IU.L-1); Δ Aspartate aminotransferase (AST) (IU.L-1). Dotted lines are normal values.

Four biochemical markers of hepatic function were tested: ALT, AST, γGT and ALP. Initially, increases of AST and ALT activities were observed, while γGT and ALP levels were normal (Figure 3). Three days after admission, serious liver damage developed, characterized by augmented AST, ALT and γGT levels. ALP level also slightly increased. Five days after hospitalization, the maximum levels reached by ALT, AST and γGT were 427, 330 and 343 IU·L^−1^ respectively. At that moment, ALP levels were normal. Several biochemical rates were calculated based on this data: γGT was 6 fold, ALT was 10 fold, AST was 9 fold and ALT/ALP was 13 fold higher than normal values. These results indicated important hepatocelullar damage without cholestatic alterations since ALP and bilirubin were normal.

AST, ALT and γGT levels still increased over the next 6 days and after that returned to normal. Twenty days after admission all enzymatic activities were normal and the patient was discharged.

### 2.4. Renal Function Parameters

After admission, creatinine and urea serum levels increased. The results showed kidney damage. The highest reached values were 2.4 mg·dL^−1^ for creatinine at admission time and 128 mg·dL^−1^ for urea at 10 h. Those parameters tended to normal values when the patient’s condition improved after 20 days.

A small collection of case reports have described a range of illnesses associated with recreational exposure to cyanobacteria. Symptoms like fever and gastrointestinal illness were the most frequently reported, but severe headache, pneumonia and myalgia were also described. The main public health concern regarding exposure to freshwater cyanobacteria, principally to *Microcystis* spp. and *Anabaena* spp. blooms, relates to the understanding that some blooms produce toxins (Microcystins and saxitoxins) that specifically affect the liver or the nervous system [8].

This report describes a case of acute poisoning by direct exposure to bioactive cyanobacterial compounds in a bloom in Salto Grande reservoir, Argentina in January 2007.

The exposure of a healthy young man to an intense cyanobacterial bloom took place by direct contact with the bloom by immersion, oral ingestion (since sudden immersion stimulates reflex swallowing) and inhalation during at least 2 h, according to what the patient describes.

Total phytoplankton analyses on samples in Salto Grande Dam were performed. Cell identification and enumeration provided evidence that *Microcystis wesenbergi* and *Microcystis aeruginosa* were the principal species found, reaching more than 33,000 cell·mL^−1^. Analyses of microcystins in study bloom samples showed the presence of microcystin-LR reaching 48 μg·L^−1^. Under these conditions short-term adverse health outcomes, e.g., skin irritations and gastrointestinal illness, are expected [10]. Even though it was not possible to discard other toxins present in the water, other associated factors such as bacteria, viruses and fungi were discarded since an atypical pneumonia was diagnosed.

Toxic cyanobacteria blooms have been identified in 13 out of 22 provinces along Argentina [11–15]. Microcystins (MCs) are hepatotoxic cyanotoxins mainly produced in La Plata Basin by the cyanobacteria *Microcystis* spp. [16–18].

These toxins are cyclic heptapeptides consisting of seven amino acids and are considered the most common group of cyanotoxins in estuaries, ponds and water reservoirs.

The primary source of human exposure to MCs is through the chronic ingestion of contaminated drinking water that may also be responsible for the increasing incidence of primary liver cancer in humans living in some areas of China [19].

However, reports of acute intoxications with MCs are exceptional. A well-known case in humans is an outbreak of severe hepatitis that occurred at a Brazilian haemodialysis centre in Caruaru (Brazil), where 100 patients developed acute liver failure and 50 of them died [20].

The absorbed MCs were taken up into the hepatocyte, and to a lesser extent to other cells, by protein transporters such as the multi-specific bile acid transporter [21]. Once in the cell, MCs induce toxicity in apparently independent ways. MCs are able to inhibit serine/threonine protein phosphatases (PP1 and PP2) which causes to cytoskeletal alterations in liver and kidney [22], depletion of glycogen stores [23], inmunosupression [24] and modify the activities of mitogen-activated protein kinases (MAPK) that support tumor-promoting activities [25]. MCs trigger the production of reactive oxygen species (ROS), which, in turn, increase the formation of lipid peroxides (LPO) [26–28] and induce mitochondrial damage, calpain release and apoptosis [29].

Nevertheless, some authors found that MCs also affect other organs such as kidney and lungs [30–32].

In this example, the presence of bioactive cyanobacterial compounds such as Microcistina-LR in the bloom coincided with the patient’s symptoms and exposure to the bloom.

The first symptoms of intoxication appeared within 4 h of the exposure ending, and then had an evolution during 20 days that we separate in three stages. An initial stage, characterized by gastrointestinal disorder, was followed by a pulmonary stage. Finally, the third stage developed as hepatotoxicosis and multiple organ failure.

Gastrointestinal disorder stage includes symptoms such as nausea, vomiting, fever, headache beginning a few hours after the exposure. These non-specific symptoms were reported, even, in non-toxic blooms in the USA, Zimbabwe, Brazil, Australia, Canada and Switzerland [10]. All of them share the same development of symptoms when being in contact with *Microcystis* spp. and *Anabaena* spp. blooms. Possible toxic agents known as bacteria, virus and parasites were discarded.

A case report concerning a man affected by a cyanobacterial bloom when he accidentally fell into the water and swallowed an estimated of 240 mL of water bloom was described by Dillinberg *et al*. [33]. In concordance with this report, within hours this person developed an acute gastrointestinal disorder that progressed to flu as illness. The pulmonary stage was described, in our case, as Idiopathic pneumonia and includes tachipnea, low PO_2_ and lung infiltration. In concordance with this, Stewart *et al*. [8] found that people who used personal watercraft on lakes with high cyanobacteria concentrations are more likely to report respiratory symptoms than people who used their personal watercraft or lakes with low cyanobacteria concentrations. Also, pneumonia was associated with cyanobacteria by [34] when an outbreak of possible cyanobacteria poisoning after contact with water containing toxic *Microcystis aeruginosa* at a freshwater reservoir in Staffordshire, England was described. In both cases the patients presented left basal pneumonia four to five days after taking part in a canoeing exercise. One (case 1) had difficulty sleeping that night and awoke the next morning with a sore throat, dry cough, vomiting and abdominal pains, and the other, developed similar symptoms within 24 h plus difficulty walking [34].

The inhalation route for exposure to cyanotoxins may be hazardous in theory, but limited experimental data supports this possibility. Direct cell contact and toxins in the alveolar surface could produce a local inflammatory reaction and make the access to the circulatory torrent of the toxins possible. It has already been demonstrated that this toxin can reach the lung after oral and intratracheal administrations as described by [35–37] which detected pulmonary thrombosis in mice intraperitoneally (i.p.) injected with lethal doses of microcystins.

The third stage of hepatotoxicosis showed in this case as hepatocellular damage is characteristic of mediated intoxication by MCs. This was described for the first time in an incident in Caruaru, when dialyzed patients were exposed to microcystins present in the net water which entered directly into their circulatory system causing hepatic damage. The hepatic damage that produces microcystins has been studied in “*in vitro*” and “*in vivo*” models, but it has not been described so far in cases of recreational poisoning. [6] The subsequent recovery patient observed in this case is concordant with the great capacity of recovery that the hepatic tissue has after a severe affection with microcystins, as has been demonstrated by Andrinolo *et al*. [38,39].

Other authors demonstrate the presence of MCs in serum samples of fishermen in Chaohu Lake, China in conjunction with rising serum enzyme levels that would indicate liver damage in populations with high levels of exposure to MCs producing cyanobacterial blooms [40].

Toxic blooms may recur periodically in surface drinking and recreational water sources; it is probable that more cases of poisoning with cyanobacteria will occur each year and that these might not be identified due to their unspecific symptoms [6] and physicians’ lack of knowledge about these kinds of poisonings.

## 3. Experimental Section

### 3.1. Sampling Site and Analysis

Salto Grande reservoir is located in Concordia (Entre Rios, Argentina). It is a subtropical lake with a surface area of 783 km^2^, a mean depth of 6.4 m. In all cases, replicate samples were collected for a quantitative phytoplankton and toxin analysis on the same day and at the same place where the patient was immersed (30°53′29″S–57°58′05″W) within 4 h of the incident.

### 3.2. Phytoplankton Analyses

Phytoplankton samples were taken from the lake with Van Dorn bottle and were fixed “*in situ*” with a 1% lugol solution for their subsequent analysis with an inverted microscope (Carl Zeiss-Axiovert 40C), using Utermöhl method [41]. Phytoplankton composition was performed according Konared and Anagnostidis [42].

### 3.3. Microcystins Determination in Water Samples

Water samples were taken in the context of a monitoring project the includes several points of the del Plata basin, some of them very distant from one another and more than 1000 km away from the laboratory. For toxin analyses, the samples were transported to the laboratory in an ice chest and stored at −20 °C. To detect MCs, the water sample (500 mL) was subjected to 3 cycles of freezing and defrosting, then filtered and finally applied to a pre-activated Sep-Pak C18 ODS (Waters). The toxins were eluted with 80% methanol and quantitative chromatographic analysis of MCs was performed by HPLC with a photodiode array detector (Shimadzu 20A) and a C18 column Thermo (5 μm pore, 150 × 4.60 mm). The column was equilibrated with a mixture composed by 65% of solution A (water with 0.05% (v/v) trifluoroacetic acid (TFA)) and 35% of solution B (acetonitrile with 0.05% (v/v) TFA). The mobile phase consisted of a discontinuous gradient of A and B solutions. The flow rate was 1.0 mL/min. MCs were identified on the basis of their UV spectra and retention time. Standards of MC-RR, MC-YR and MC-LR were purchased from Sigma (St Louis, MO, USA).

### 3.4. Biochemical Parameters

When the patient was admitted to hospital, arterial and venous blood samples were collected by trained health service staff in order to determine hemogram, ionogram, acid-base status, liver and renal function.

### 3.5. Ionogram and Acid-Base Status Parameters

In heparinized arterial blood samples PO_2_, PCO_2_, SatHb, Na^+^, K^+^, HCO_3_ ^−^ and Cl^−^ were periodically measured by Stat Profile PHOX Plus L (NOVA Biomedical) and Electrolite Analyzer (NOVA Biomedical) during patient hospitalization.

### 3.6. Liver and Renal Function Parameters

Ten milliliters of blood samples were periodically collected by venipuncture and left to clot. The clotted blood was centrifuged 15 min at 1500 g to yield serum. The following parameters were measured in serum samples: alanine aminotransferase (ALT), aspartate aminotransferase (AST), alkaline phosphatase (ALP), γ-glutamyltransferase (GGT), lactate dehydrogenase (LDH), total bilirubin (TBIL), direct bilirubin (DBIL), indirect bilirubin (IBIL), glucose (Glu), Urea (U) and Creatinin (Cre). Serum determinations were performed by KONELAB 30i (Wiener).

### 3.7. Hemogram

Hemogram was performed by CELL-DYN Ruby (Abbott), in venous blood sample with EDTA.

## 4. Conclusion

An acute case of intoxication with cyanobacterial and cyanotoxins in recreational water in Salto Grande dam, Argentina is reported and linked with an intense bloom of *Microcystis wesenbergii* and *Microcystis aeruginosa* with a presence of 48.6 μg·L^−1^ of microcystin-LR in water samples.

In this acute case of intoxication, an initial stage, characterized by gastrointestinal disorder, was followed by a pulmonary stage and the third stage was initiated as hepatotoxicosis and multiorgan failure was observed in the patient.

This is the first study that shows an acute intoxication with cyanobacterial compounds in blooms in recreational water.

It is important to develop cyanotoxicosis detection programs that may include training of health professionals, the development of specific MCs exposure biomarkers and regular environmental monitoring, aiming to establish the degree of human exposure to cyanotoxins, to properly diagnose poisonings, even the mild ones, and to establish criteria for risk management.

## References

[1] Codd G.A., Lindsay J., Young F.M., Morrison L.F., Metcalf J.S., Huisman J., Matthijs H.C.P., Visser P.M. (2005). Harmful Cyanobacteria. From Mass Mortalities to Management Measures. Harmful Cyanobacteria.

[2] MacKintosh C., Beattie K.A., Klumpp S., Cohen P., Codd G.A. (1990). Cyanobacterial microcystin-LR is a potent and specific inhibitor of protein phosphatases 1 and 2A from both mammals and higher plants. FEBS Lett.

[3] Gulledgea B.M., Aggena J.B., Huangb H.B., Nairnc A.C., Chamberlin A.R. (2002). The microcystins and nodularins: cyclic polypeptide inhibitors of PP1 and PP2A. Curr. Med. Chem.

[4] van Apeldoorn M.E., van Egmond H.P., Speijers G.J., Bakker G.J. (2007). Toxins of cyanobacteria. Mol. Nutr. Food Res.

[5] Carmichael W.W., Falconer I.R., Falconer I.R. (1993). Diseases Related to Freshwater Blue Green Algal Toxins, and Control Measures. Algal Toxins in Seafood and Drinking Water.

[6] Backer L., McNeel S.W., Barber T., Kirkpatrick B., Williams C., Irvin M., Zhou Y., Johnson T.B., Nierenberg K., Aubel M. (2010). Recreational exposure to microcystins during algal blooms in two California lakes. Toxicon.

[7] Echenique R., Sedán D., Giannuzzi L., Niez-Gay A., Andrinolo D (2009). Impacto producido por floraciones de Cyanobacteria en agua de red (Concordia, Entre Ríos). XXXII Jornadas Argentinas de Botánica.

[8] Stewart I.P., Webb M., Schluter P.J., Shaw G.R. (2006). Recreational and occupational field exposure to freshwater cyanobacteria—a review of anecdotal and case reports, epidemiological studies and the challenges for epidemiologic assessment. Environ. Health.

[9] Backer L., Carmichael W., Kirkpatrick B., Williams C., Irvin M., Zhou Y., Johnson T.B., Nierenberg K., Hill V.R., Kieszak S.M. (2008). Recreational exposure to low concentrations of Microcystins during an algal bloom in a small lake. Mar. Drugs.

[10] Kuiper-Goodman T., Falconer I., Fitzgerald J, Chorus I., Bartram J. (1999). Human Health Aspects. Toxic Cyanobacteria in Water. A Guide to Their Public Health Consequences, Monitoring and Management.

[11] Amé M., Díaz M., Wunderlin D. (2003). Occurrence of toxic cyanobacterial blooms in San Roque Reservoir (Córdoba, Argentina): A field and chemometric study. Environ. Toxicol.

[12] Ruibal Conti A.L., Guerrero J.M., Regueira J.M. (2005). Levels of microcystins in two Argentinean reservoirs used for water supply and recreation: differences in the implementation of safe levels. Environ. Toxicol.

[13] Cazenave J., Wunderlin D.A., Bistoni M.A., Ame M.V., Krause E., Pflugmacher S., Wiegand C. (2005). Uptake, tissue distribution and accumulation of Microcystin-RR in *Corydoras paleatus*, *Jenynsia multidentata* and *Odontesthes bonariensis*. Aquat. Toxicol.

[14] Andrinolo D., Pereira P., Giannuzzi L., Aura C., Massera S., Caneo M., Caixach J., Barco M., Echenique R. (2007). Occurrence of *Microcystis aeruginosa* and microcystins in Rio de la Plata river (Argentina). Acta Toxicol. Argent.

[15] Ame M.V., Galanti L., Menone M., Gerpe S., Moreno V., Wunderlin D. (2010). Microcystin-LR, -RR, -YR and -LA in water samples and fishes from a shallow lake in Argentina. Harmful Algae.

[16] De Leon L., Yunes J. (2001). First report of a *Microcystis aeruginosa* toxic bloom in La Plata river. Environ. Toxicol.

[17] Ouahid Y., Zaccaro M., Zulpa G., Storni M., Stella A., Bossio J.C., Tanuz M., del Campo F. (2011). A single microcystin in a toxic Microcystis bloom from the river Rio de Plata, Argentina. Int. J. Environ. Anal. Chem.

[18] Echenique R., Rodríguez J., Caneo M., Gianuzzi L., Barco M., Rivera J., Caixach J., Andrinolo D (2006). Microcystins in the Drinking Water Supply in the Cities of Ensenada and La Plata (Argentina).

[19] Ueno Y., Nagata S., Tsutsumi T., Hasegawa A., Watanabe M.F., Park H.D., Chen G.C., Chen G., Yu S.Z. (1996). Detection of microcystins, a blue-green algal hepatotoxin, in drinking water sampled in Haimen and Fusui, endemic areas of primary liver cancer in China, by highly sensitive immunoassay. Carcinogenesis.

[20] Jochimsen E.M., Carmichael W.W., An J.S., Cardo D.M., Cookson S.T., Holmes C.E.M., Antunes M.B., de Melo Filho D.A., Lyra T.M., Barreto B.S.T. (1998). Liver failure in death after exposure to microcystins at a hemodialysis center in Brazil. N. Engl. J. Med.

[21] Bury N.R., Newlands A.D., Eddy F.B., Codd G.A. (1998). *In vivo* and *in vitro* intestinal transport of 3H-microcystin-LR, a cyanobacterial toxin, in rainbow trout (*Oncorhynchus mykiss*). Aquat. Toxicol.

[22] Solter P., Wollenberg G., Huang X., Chu F., Runnegar M. (1998). Prolonged sublethal exposure to the protein phosphatase inhibitor MC-LR Results in multiple dose-dependent hepatotoxic effects. Toxicol. Sci.

[23] Guzman R.E., Solter P.F. (2002). Characterization of sublethal microcystin-LR exposure in mice. Vet. Pathol.

[24] Chen T., Zhao X., Liu Y., Shi Q., Hua Z., Shen P. (2004). Toxicology analysis of immunomodulating nitric oxide, iNOS and cytokines mRNA in mouse macrophages induced by microcystin-LR. Toxicology.

[25] Pahan K., Sheikh F., Namboodiri A., Singh I. (1998). Inhibitors of protein phosphatase 1 and 2A differentially regulate the expression of inducible nitric-oxide synthase in rat astrocytes and macrophages. J. Biol. Chem.

[26] Guzman R.E., Solter P.F. (1999). Hepatic oxidative stress following prolonged sublethal microcystin LR exposure. Toxicol. Pathol.

[27] Moreno I., Pichardo S., Jos A., Gómez-Amores L., Mate A., Vazquez C.M., Cameán A.M. (2005). Antioxidant enzyme activity and lipid peroxidation in liver and kidney of rats exposed to MC-LR administered intraperitoneally. Toxicon.

[28] Andrinolo D., Sedan D., Telese L., Aura C., Masera S., Giannuzzi L., Marra C., Alaniz M.T. (2008). Recovery after damage produced by subchronic intoxication with the cyanotoxin microcystin LR. Toxicon.

[29] Ding W., Ong C. (2003). Role of oxidative stress and mitochondrial changes in cyanobacteria-induced apoptosis and hepatotoxicity. FEMS Microbiol. Lett.

[30] Nobre A.C.L., Jorge M.C.M., Menezes D.B., Fonteles M.C., Monteiro H.S.A. (1999). Effects of microcystin-LR in isolated perfused rat kidney. Braz. J. Med. Biol. Res.

[31] Dias E., Andrade M., Alverca E., Pereira P., Batoreu M.C., Jordan P., Silva M.J. (2009). Comparative study of the cytotoxic effect of microcistin-LR and purified extracts from *M. aeruginosa* on a kidney cell line. Toxicon.

[32] Soares R.M., Cagido V.R., Ferraro R.B., Meyer-Fernandes J.R., Rocco P.R., Zin W.A., Acevedo S.M. (2007). Effects of microcystin-LR on mouse lungs. Toxicon.

[33] Dillinberg H.O., Dehnel M.K. (1960). Toxic waterbloom in Saskatchewan, 1959. Can. Med. Assoc. J.

[34] Turner P.C., Gammie A.J., Hollinrake K., Codd G.A. (1990). Pneumonia associated with cyanobacteria. Br. Med. J.

[35] Ito E., Kondo F., Harada K. (2000). First report on the distribution of orally administered microcystin-LR in mouse tissue using an immunostaining method. Toxicon.

[36] Ito E., Kondo F., Harada K. (2001). Intratracheal administration of microcystin-LR and its distribution. Toxicon.

[37] Slatkin D.N., Stoner R.D., Adams W.H., Kycia J.H., Siegelman H.W. (1983). Atypical pulmonary thrombosis caused by a toxic cyanobacterial peptide. Science.

[38] Andrinolo D., Sedán D., Telese L., Aura C., Masera S., Giannuzzi L., Marra C.A., Alaniz M. (2008). Hepatic recovery after damage produced by sub-chronic intoxication with the cyanotoxin microcystin-LR. Toxicon.

[39] Lance E., Josso C., Dietrich D., Ernst B., Paty P., Senger F., Bormans M., Gérard C. (2010). Histopathology and microcystin distribution in *Lymnaea stagnalis* (Gastropoda) following toxic cyanobacterial or dissolved microcystin-LR exposure. Aquat. Toxicol.

[40] Chen X., Xie P., Li L., Xu J. (2009). First Identification of the hepatotoxic microcystins in the serum of a chronically exposed human population together with indication of hepatocellular damage. Toxicol. Sci.

[41] Utermöhl H. (1958). Zur Vervolkommung der quantitative Phytoplankton-Methodik. Mitt. Int. Verein. Limnol.

[42] Komárek J., Anagnostidis K, Ettl H., Gärtner G., Heyning H., Mollenhauer D. (1999). Sübwasserflora von Mitteleuropa.

